# Exhaustively characterizing feasible logic models of a signaling network using Answer Set Programming

**DOI:** 10.1093/bioinformatics/btt393

**Published:** 2013-07-12

**Authors:** Carito Guziolowski, Santiago Videla, Federica Eduati, Sven Thiele, Thomas Cokelaer, Anne Siegel, Julio Saez-Rodriguez

**Affiliations:** ^1^École Centrale de Nantes, IRCCyN UMR CNRS 6597, 44321, Nantes, France, ^2^CNRS, UMR 6074 IRISA, Campus de Beaulieu, 35042 Rennes, France, ^3^INRIA, Dyliss project, Campus de Beaulieu, 35042 Rennes, France, ^4^Universität Potsdam, Institut für Informatik, D-14482 Potsdam, Germany and ^5^European Molecular Biology Laboratory, EuropeanBioinformatics Institute, Hinxton CB10 1SD, UK

## Abstract

**Motivation:** Logic modeling is a useful tool to study signal transduction across multiple pathways. Logic models can be generated by training a network containing the prior knowledge to phospho-proteomics data. The training can be performed using stochastic optimization procedures, but these are unable to guarantee a global optima or to report the complete family of feasible models. This, however, is essential to provide precise insight in the mechanisms underlaying signal transduction and generate reliable predictions.

**Results:** We propose the use of Answer Set Programming to explore exhaustively the space of feasible logic models. Toward this end, we have developed *caspo*, an open-source Python package that provides a powerful platform to learn and characterize logic models by leveraging the rich modeling language and solving technologies of Answer Set Programming. We illustrate the usefulness of *caspo* by revisiting a model of pro-growth and inflammatory pathways in liver cells. We show that, if experimental error is taken into account, there are thousands (11 700) of models compatible with the data. Despite the large number, we can extract structural features from the models, such as links that are always (or never) present or modules that appear in a mutual exclusive fashion. To further characterize this family of models, we investigate the input–output behavior of the models. We find 91 behaviors across the 11 700 models and we suggest new experiments to discriminate among them. Our results underscore the importance of characterizing in a global and exhaustive manner the family of feasible models, with important implications for experimental design.

**Availability:** *caspo* is freely available for download (license GPLv3) and as a web service at http://caspo.genouest.org/.

**Supplementary information:** Supplementary materials are available at *Bioinformatics* online.

**Contact:** santiago.videla@irisa.fr

## 1 INTRODUCTION

Predictive models of biological networks are a main component of systems biology. For a certain system of interest, if enough information is available about the biomolecules that constitute it and their interactions, one can convert this prior knowledge into a mathematical model (e.g. a set of differential equations or logic rules) that can be simulated. If experimental data are available, the model can be fitted (trained) to the data. That is, one determines the model parameters (for example, kinetic constants in a biochemical model) to obtain the most plausible model given the data. This is normally achieved by defining an objective function that describes the goodness of the model based on the data that is subsequently optimized (Banga, 2008).

This training process is not a trivial task owing to factors including experimental error, limitations in the amount of data available, incompleteness of our prior knowledge and inherent mathematical properties of the models. Thus, in general, there is no single solution but rather multiple models that describe the data equally (or similarly) well. In those cases, the model is said to be non-identifiable (Kreutz and Timmer, 2009; Walter and Pronzato, 1996).

In some cases, deterministic methods that guarantee the identification of the optimal models can be applied, but these methods are often limited by the exponential growth of the search space. Thus, usually one needs to use stochastic methods that may identify the optimum or at least exhibit suboptimal models (Banga, 2008). However, an incomplete characterization of the set of plausible models limits significantly the insight that can be gained about the underlying molecular mechanisms.

In this article, we investigate this issue in the context of logic modeling of signaling networks. These models have been applied recently to analyze signal transduction in a variety of contexts (Calzone *et al.*, 2010; Wang *et al.*, 2012). In particular, given a network encoding our knowledge of signal transduction and a dataset measuring the activation of proteins in this network on various perturbations, one can derive from the network (Boolean) logic models fitted to the data. Models are simulated assuming that the network reaches a pseudo steady state at a certain time on stimulation, and the identification of the network that best fits the data is posed as an optimization problem. This problem can be solved using meta-heuristics (e.g. a genetic algorithm), and their application suggests that there are multiple alternative models that explain the data (Saez-Rodriguez *et al.*, 2009). However, stochastic search methods cannot characterize the models precisely: they are intrinsically unable not just to provide a complete set of solutions, but also to guarantee that an optimal solution is found. To overcome this limitation, approaches based on Integer Linear Programming (ILP) (Mitsos *et al.*, 2009; Sharan and Karp, 2012) and Answer Set Programming (ASP) (Videla *et al.*, 2012) have been applied, providing a proof of concept that a global optimum can be identified.

Here we present *caspo*, a free open-source tool to learn (Boolean) logic models of signal transduction in a complete and global fashion. *caspo* uses CellNOpt pre- and post-processing routines [Terfve *et al.* (2012)]. It can handle feedback loops in the prior knowledge network (PKN), numerical datasets and tolerance in the score owing to experimental uncertainty. We use *caspo* to exhaustively explore the space of optimal and suboptimal models for a real case describing pro-growth and inflammatory pathways in a liver cancer cell. We find that, even with small tolerance, thousands of models can be compatible with the data and use ASP’s flexibility to further analyze them: we categorize them according to their input–output behavior and identify subsets of modules that are interchangeable with respect to the score. The multiple possible combinations of these modules are responsible for the large number of models found.

## 2 METHODS

### 2.1 Learning Boolean logic models

Our prior knowledge about signal transduction can be described as a set of causal interactions among the biomolecules involved (mostly proteins) that can be mathematically formulated as a signed and directed graph. We call this graph the PKN. In such a graph, one can denote as *input* nodes those that can be stimulated or inhibited experimentally. When the system is perturbed by fixing the state of such nodes, one can measure the activity of each *output* node being observed. Such measurements are typically given by *phospho-proteomics datasets* consisting of measurements over *m* proteins under *n* experimental conditions. With 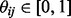, we denote the activity of a protein *j* under the experimental condition *i*, where 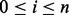 and 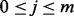. In agreement with experimental errors, we used a discretization procedure so that 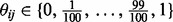.

The state of nodes after a perturbation of the system cannot be predicted using only graph theory. However, a simple framework is given by Boolean logic models (Klamt *et al.*, 2006). In a logic model, activation of nodes is defined by a set of operators. We use the representation known as sum of products (SOP; also called disjunctive normal form), which uses only AND (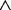), OR (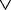) and NOT (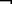) operators. A simple form to encode logic models based on the SOP formalism is using hypergraphs (Klamt *et al.*, 2006). A directed and signed *hypergraph* 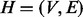 is a generalization of a directed and signed graph 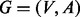, where *V* is the set of nodes and *E* the set of *hyperedges*. While edges in *A* connect pairs of nodes 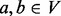, *hyperedges* in *E* connect pairs of *sets of nodes* 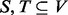. To describe a logic model as a hypergraph, each SOP expression is mapped to a set of hyperedges.

The PKN is first compressed to simplify the structure (Saez-Rodriguez *et al.*, 2009). Then, because the exact logic gates are often not known, we perform an *expansion* to generate all possible gates compatible with the PKN. Mathematically, we derive a hypergraph 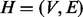 from a graph 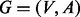, so that for every signed hyperedge 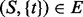 and every 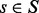, there exists an edge 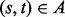 having the corresponding sign.

Let *H* be a hypergraph describing a logic model and 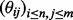 be a phospho-proteomics dataset. For each experimental condition *i*, we can compute the Boolean prediction 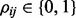 of the state of a protein *j* by using the logic formulas described by *H*. This corresponds to computing the (quasi) steady state of the system. These simulated values at a quasi steady state are considered an approximation of the state of the cell immediately after a perturbation and can be thus compared with experimental values obtained at early times after stimulation (Klamt *et al.*, 2006).

Then, the *fitness of the logic model* to the experimental dataset is obtained by comparing experimental observations, normalized between 0 and 1, with Boolean predictions based on the mean square error (MSE) as follows: 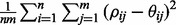.

*Combinatorial optimization problem.* The problem of learning Boolean logic models that we address in this work consists of finding minimal hypergraphs derived from the PKN that minimize the MSE where the size of a hypergraph *H* is the sum of cardinalities of each hyperedge source (i.e. the sum of the number of inputs): 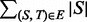. Thus, the problem can be formulated as a lexicographic multi-objective optimization where the first objective is to minimize MSE, and the second objective is to minimize size. Our prior assumption that 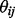 belongs to a finite set of values implies that this problem is of discrete nature. Further, the optimization can be relaxed by using different degrees of tolerance over the optimum for each objective, i.e. MSE and size.

*Global Truth Tables.* Inspired by truth tables in propositional logics, we introduce the concept of Global Truth Tables (GTTs) as a way of describing the input–output behavior of a Boolean logic model. For a given logic model, we can compute its predictions on observable *output* nodes in response to every possible experimental condition on *input* nodes. Comparing GTTs allows one to decide whether two logic models, regardless of their structures, are experimentally distinguishable. Furthermore, GTTs provide a way of grouping a large number of logic models according to their input–output behavior to facilitate the analysis.

### 2.2 Learning Boolean logic models with ASP

ASP is a declarative problem-solving paradigm from the field of Logic Programming combining several computer science areas (Baral, 2003; Gebser *et al.*, 2013). As a full declarative paradigm, instead of telling a computer *how to solve the problem*, with ASP one defines *what the problem is* and leaves its solution to the solver. These solvers are based on Boolean constraint solving technology, and they can solve hard discrete combinatorial search problems, with comparable results with ILP.

The distinct feature of ASP is its rich modeling language, making it popular as a tool for declarative problem solving. Sophisticated pre-processing techniques (*grounding*) are required for dealing with this rich language. Thanks to the development of an ASP language standard, its expressiveness and powerful solvers, ASP has been widely used in many fields of computer science for a decade. Recently, the capability of solvers has increased such that ASP started to be applied to solve hard combinatorial problems arising in bioinformatics and systems biology. Applications include expanding metabolic networks (Schaub and Thiele, 2009), repairing inconsistencies in gene regulatory networks (Gebser *et al.*, 2010), modeling the dynamics of regulatory networks (Fayruzov *et al.*, 2009), inferring functional dependencies from time-series data, (Durzinsky *et al.*, 2011), integrating gene expression with pathway information (Papatheodorou *et al.*, 2012) and analyzing the dynamics of reactions networks (Ray and Soh, 2012).

We used the freely available ASP grounder *gringo* and solver *clasp*, both included in the Potsdam Answer Set Solving Collection (http://potassco.sourceforge.net/). Importantly, we relied on the capability of the solvers to handle multi-criteria optimization to guarantee the global optimum by reasoning over the complete space of solutions. Several reasoning modes (enumeration, union and intersection) were also necessary to complete the combinatorial study of the family of feasible solutions. We refer the reader to the Supplementary Material for more details.

### 2.3 Software: caspo

**Fig. 1.**
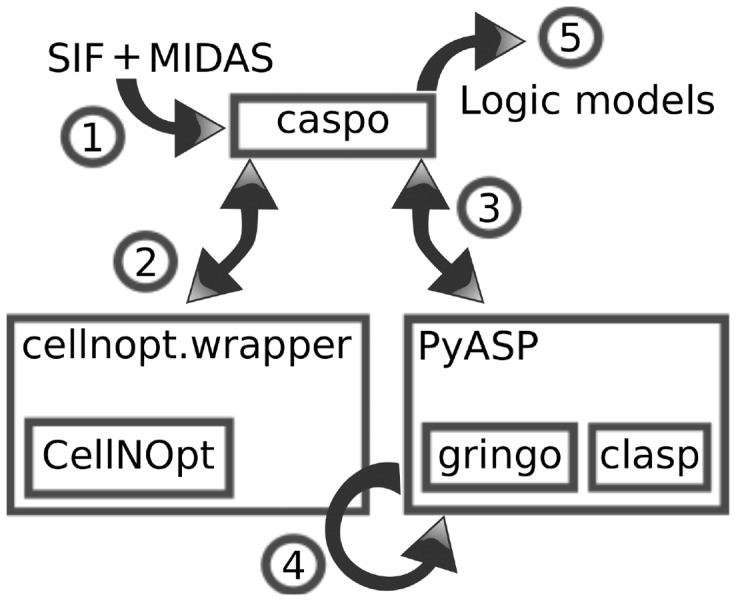
High-level design of caspo. (1) Input files are a PKN in Cytoscape’s SIF format, and a dataset as a CSV file in the MIDAS format (Supplementary Material). (2) Pre-processing routines by CellNOpt. (3) Finds an optimum model. (4) Finds all models within the tolerance. (5) Outputs all models found

We have implemented *caspo*: *Cell ASP Optimizer*, a Python package that combines PyASP (http://pypi.python.org/pypi/pyasp) and CellNOpt (http://www.cellnopt.org/) to provide an easy -to-use software for learning Boolean logic models (Fig. 1). The software is freely available for download and also as a web service through the Mobyle framework (Néron *et al.*, 2009). PyASP encapsulates the main ASP tools, *gringo* and *clasp*, into Python objects. These objects can be fed with logic programs describing different tasks, be launched with dedicated parameter settings and return the ASP results for further processing. CellNOpt [Terfve *et al.* (2012)] is a software for training logic models using different formalisms (Boolean, Fuzzy or Ordinary Differential Equations). The software allows us to import and pre-process a PKN, normalize experimental data, train logic models to data using heuristic methods and post-process and visualize the resulting models. CellNOpt is written as a set of R packages available on Bioconductor and as a Cytoscape plugin (CytoCopter), and it can used within Python using the package *cellnopt.wrapper*.

## 3 RESULTS

To illustrate the use of *caspo*, we use a model of pro-growth and pro-inflammatory model in liver cells. The model is trained to phospho-proteomics data generated in the liver cancer cell line HepG2. Data are generated on perturbation with combination of ligands and small-molecule inhibitors blocking the activities of specific kinases (Alexopoulos *et al.*, 2010). The dataset contains measurements using the Luminex technology of 15 species under 64 experimental conditions. This model was introduced in (Saez-Rodriguez *et al.*, 2009) and here we use a variation from (Morris *et al.*, 2011). In this case, there are 130 possible hyperedges and thus, the number of possible logic models (i.e. search space of the combinatorial optimization) is given by 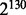.

### 3.1 Family of optimal models

We first used *caspo* to compute all global optimum solutions to the optimization over our case study. We found 16 Boolean logic models (Supplementary Fig. S1) with minimal score (0.36 s), all models having the same fitness to data (MSE = 0.0499) and size (28). Moreover, the same 16 logic models were found (0.5 s) using an extended PKN with feedback loops from Terfve *et al.* (2012). Cross validation analysis showed no significant difference in the optimum MSE with respect to the complete dataset (Supplementary Fig. S2).

The 16 different models arise owing to four pairs of submodels (modules) equivalent in terms of score. These modules represent alternative ways to activate specific nodes and are independent from each other. For each pair, only one of the modules appears in a given model; that is, they are *mutually exclusive*. Thus, selecting either member of each pair provides an optimal model and all possible combinations give rise to the 2^4 ^= 16 models. To elucidate the differences between the 16 models from their responses to all possible experimental conditions, we computed and compared their GTTs (Section 2.1). Interestingly, they all have the same GTT. That is, for any combination of input nodes (stimuli and inhibitors), the same values are predicted for all the readouts by the 16 models. Therefore, the optimization reports a single solution in terms of input–output behavior, despite the fact that this solution can take the form of any of the 16 models. To distinguish among these models (and thus determine which of the mutually exclusive modules are functional), we would require a different experimental setup, i.e. new species have to be either perturbed or measured.

### 3.2 Suboptimal Models: Enumeration and Structure

**Fig. 2.**
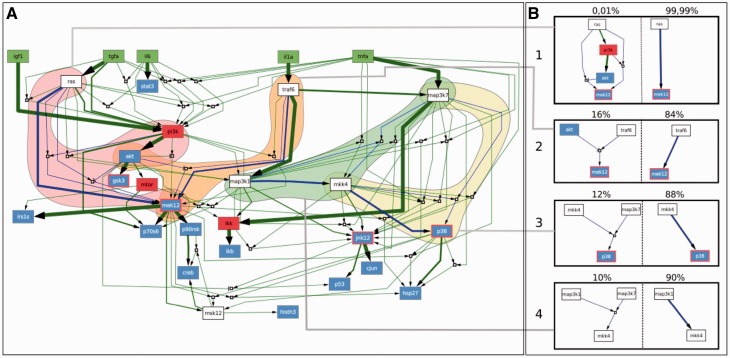
Suboptimal models generated with *caspo* with 10% error tolerance. (**A**) Network of the union of 11 700 suboptimal models. Green nodes represent ligands that are experimentally stimulated. Red (or red-bordered) nodes represent those species that are inhibited with a small molecule inhibitor (drug). Blue nodes represent species that were measured using the Luminex technology. White nodes are neither measured nor perturbed. AND gates in the models are represented by empty boxes. The thickness of the hyperedges correspond to their frequencies among the 11 700 submodels. (**B**) Four pairs of mutually exclusive modules (blue hyperedges in A) and their corresponding frequencies on top. These modules determine the behavior of three nodes in the network: mek12, mkk4 and p38

Experimental error is inherent in biochemical data. Therefore, one needs to consider models whose predictions deviate from those of the optimal one by an amount within the experimental error (Saez-Rodriguez *et al.*, 2009). Considering that the optimization minimize MSE and size, we defined as *suboptimal models* those solutions having MSE within a 10% of tolerance with respect to the MSE of optimal models (a conservative approximation to the real experimental error), and maximal size of 28 (the size of the optimal models; Section 3.1). From these settings, *caspo* found 11 700 suboptimal models (Fig. 2) with sizes 28, 27, 26 and 25 whose MSE spanned from 0.0499 to 0.0546. We observed that the number of models decreases exponentially with the tolerance over the MSE (e.g. 8%—7378 models, 6%—6048 models, 2%—192 models). Allowing also a tolerance over the size would generate a much larger number of models by the addition of spurious links to those of size 28 (e.g. size 29–51 480 models, size 30–189 364 models). We therefore limited, for simplicity of this study, the size to 28.

**Fig. 3.**
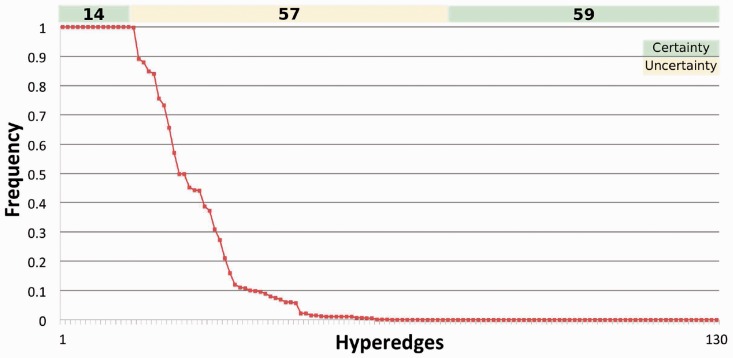
Frequencies of hyperedges over 11 700 suboptimal models within 10% tolerance. Among the 130 possible hyperedges, 14 were always present, 59 were always absent and 57 were present in some but not all models

The complete computation of suboptimal models allows a precise characterization of the distribution of hyperedges, and, therefore, of logical gates in the potential models. When we evaluated the distribution of the 130 possible hyperedges (i.e. those that are included in the hypergraph derived from the original PKN) across the 11 700 models, we found that 14 hyperedges are *present in all* suboptimal models, and we thus expect them to be functional in HepG2 cells. Fifty-nine hyperedges are *absent from all models*, thus suggesting that they are not functional in these cells. Finally, 57 hyperedges are present in only a subset of the models; their frequency ranges from 0.99 to 0.0003, showing a large variability (Fig. 3). Therefore, for the given experimental data, these hyperedges are not identifiable, as it is not possible to determine whether they are functional in HepG2 cells.

Analogously to the set of optimal models, we investigated the combinatorics within the family of suboptimal models. We found four mutually exclusive pairs of modules (Fig. 2B). Replacing a module of each pair by the other has no effect on the MSE for two of the pairs (1, 2 in Fig. 2B). However, for the pairs 3 and 4 there is a difference; 32 and 26.8%, respectively, of the suboptimal models differ in the output for a range from 8 to 15% of the experimental conditions. All modules were constituted by a single hyperedge, except 1A, which is set by two hyperedges: 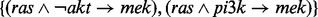 (Fig. 2, module 1A). These two hyperedges were therefore always either both present or both absent (*mutually inclusive*). As expected, there is a clear difference between the frequencies in each pair of exclusive patterns where smaller or simpler hyperedges are always more abundant. Importantly, the mutually exclusive modules for the family of suboptimal models are not the same as those present when only optimal models are considered. This indicates that the combinatorics exhibited within optimal models are not so important when considering experimental error, probably owing to the larger variability among suboptimal models.

### 3.3 Input–output behavior

To further characterize the family of suboptimal models, we next studied its input–output behavior as expressed by its GTTs. Using *caspo*, we found that the 11 700 suboptimal models correspond to 91 different GTTs. In these 91 GTTs, the predicted values are the same for 30% (4915 out of 16 384) of all the possible experimental conditions (i.e. 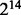 combinations of the 14 inputs of the model). Therefore, such predictions can be seen as the ‘core’ predictions of the system behavior independently from experimental noise. Considering the remaining 70% of experimental conditions, we found that at least seven experiments are needed to discriminate among all GTTs (Table S4). By performing such experiments, one would be able to generate at least one different output prediction between every pair of GTTs.

**Fig. 4.**
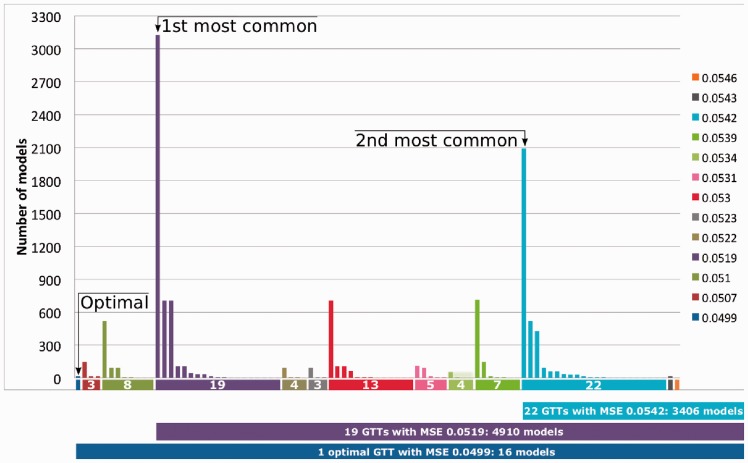
Distribution of suboptimal models. The suboptimal models are ordered (from left to right) first according to their MSEs, and then according to their 91 GTTs. The number of different models leading to the same GTT is plotted in vertical bars. GTTs are ordered and colored by their MSE. The 16 optimal models correspond to MSE 0.0499. The two most common GTTs describe the response of 3126 and 2090 models

**Fig. 5.**
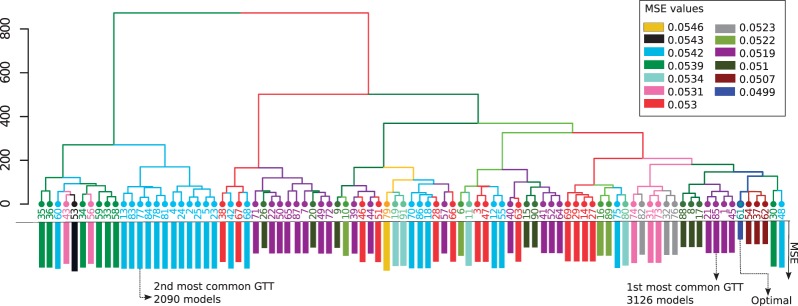
Hierarchical clustering of GTTs. Hierarchical clustering of the 91 GTTs based on their predictions for the readouts across all experimental conditions. Bars length on the leafs represents the corresponding MSE value for each GTT. The optimal GTT (61) is highlighted, as well as the two most common ones (85 and 77). The most common GTT is close to the optimal one, whereas the second most common GTT has a different behavior

Among the 11 700 suboptimal models, there are only 13 different MSEs. The distribution of such MSEs is inhomogeneous, and two MSEs (0.0519 and 0.0542) gather 71% of suboptimal models (Fig. 4). For both most frequent MSEs, a GTT is much more common than all the others: the first GTT, at MSE 0.0519, is shared by 3126 (27%) suboptimal models, while the second most common GTT, at MSE 0.0542, is shared by 2090 (18%) models. In contrast, the minimal GTT, at MSE 0.0499, was shared by only the 16 minimal models. This analysis suggests that the single optimal GTT at MSE 0.0499 is far from being representative over the 11 700 suboptimal models (0.1%). The two most common GTTs are arguably much more relevant. Interestingly, a hierarchical clustering reveals that these two most common GTTs cluster separately and that the GTT representing 27% of all suboptimal models is close to the optimal one (Fig. 5).

**Fig. 6.**
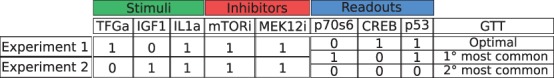
Experiments to discriminate more relevant GTTs. Both experiments generate the same output in each GTT. Stimuli not shown are inactive, inhibitors not shown are absent and readouts not shown have the same value inthree GTTs

Finally, we have investigated the space of experiments to identify the simplest ones (i.e. minimal number of stimulations and inhibitions), which maximize the pairwise differences between the optimal and the two most common GTTs. These three GTTs differ pairwise in either one or two readouts among 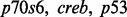, and only 192 experiments generate two differences. Out of these 192 experiments, we identified eight experiments with minimal number of stimulations, and among them, we selected the ones with minimal number of inhibitions (Fig. 6). We noted that the two experiments found generate the same output over the readouts. Thus, in contrast to the seven experiments needed to discriminate among all GTTs, only one experiment is required to discriminate between the optimal and the two most common GTTs.

### 3.4 Comparison with an stochastic optimization

We compared *caspo* with CellNOpt (Terfve *et al.*, 2012), the existing tool to solve the same problem, but using a genetic algorithm. Stochastic search methods, such as genetic algorithms, are intrinsically unable not just to provide a complete set of solutions, but also to guarantee that an optimal solution is found. Typically, one needs to combine solutions from multiple runs to increase the confidence. Thus, to illustrate the value of *caspo* in comparison with CellNOpt, we have performed multiple runs of it over the same case study.

From multiple independent runs of CellNOpt (1000 runs with an average of 1000 s per run), we found 4706 suboptimal models out of the 11 700 models found using *caspo* (70 s). The MSEs of models found with CellNOpt spanned from 0.0499 to 0.0543 (Supplementary Fig. S3). This family of models was found combining 20% of the runs, whereas in the other 80% all models found were out of the allowed tolerance range. Notably, the 16 optimal models (MSE = 0.0499) were found by CellNOpt. Concerning GTTs, the 4706 models exhibit 51 input–output behaviors out of the 91 we found with *caspo* (Supplementary Fig. S4). The genetic algorithm retrieved all the GTTs in both extremes of the hierarchical cluster, while the rest of the cluster was not completely explored (Supplementary Fig. S5). Thus, plausible behaviors away from the most common ones appear less likely to be found. These results show the relevance of a software tool like *caspo*, which allows us to explore exhaustively the space of feasible solutions in short time.

## 4 CONCLUSION

A useful approach to model large-scale signaling networks consists on training Boolean logic models from prior knowledge and dedicated experimental data. The problem of training these models is an optimization task that can be solved with stochastic search methods (Saez-Rodriguez *et al.*, 2009), which have the important limitation that they do not guarantee global optimality nor an exhaustive solution. In this article, we show how recasting this problem in a highly declarative language allows us to identify the complete family of feasible models and query them to obtain insight into model degeneracy.

In a real-case study, we have seen that there is a family of feasible models with a deep combinatorial structure: several combinations of internal submodules, with equal or similar scores, can equivalently explain the observed behavior of the system. This leads to a rapid growth of the family of suboptimal models. Taking into account the inherent noise in data, we showed that 11 700 different models can be considered as plausible representations of the PKN and an experimental phospho-proteomics dataset. Thanks to our exhaustive characterization of these models, we could determine unambiguously which hyperedges (biological links) are functional, based on their distributions across the models and determine whether groups of hyperedges are exclusive from each other.

To further characterize this family of models, we introduced the concept of GTTs and used it to explore their input–output behavior. Compared with the model topologies, the variability is much lower; the 11 700 models can be grouped in 91 GTTs, and for 30% of the 16 384 possible perturbations, all models gave the same predictions. Interestingly, the distribution of models among GTTs is far from being equidistributed, and two GTTs comprise almost half of the models, while the GTT corresponding to the optimal score is specific (0.1% of the models). While the most common GTT is similar to the GTT with optimal score, the second most common GTT is different. However, a single experiment is able to discriminate these models.

These results underscore the importance of exploring exhaustively the family of models and take into account experimental error to obtain an adequate picture of the feasible model solutions. Our formal approach based on ASP allows a precise characterization of the information that can be inferred from the confrontation of prior knowledge with experimental observations over protein signaling networks. It also permits the study of the internal combinatorics leading to the variability of the system functioning and provides a tool toward experimental design. Owing to the complexity of signaling networks and the limitations of existing experimental technologies (in terms of which nodes can be measures and/or perturbed), models typically show an important lack of identifiability. This is a general limitation of models in systems biology (Kreutz and Timmer, 2009). In the context of Boolean models, we expect that further development of experimental design (Sharan and Karp, 2012), in intimate coordination with advances in experimental techniques will allow us to tackle this issue.

This work opens the way to several prospective tracks. First, it would be useful to evaluate our ASP formulation and those based on ILP from (Mitsos *et al.*, 2009) and (Sharan and Karp, 2012) to understand their strengths and complementary features. In contrast to ILP, ASP is a relatively new tool for problem solving in biology. ASP, having its roots in knowledge representation and reasoning, has proven to be well suited to address highly combinatorial search and discrete optimization problems, with at least comparable performance with well established ILP solvers. On the other hand, ILP as a mathematical programming framework may be more suitable to study problems based on calculus over large domains of integer or rational numbers. Therefore, combining the expressiveness and power of several solving technologies instead of selecting one of them seems a promising option for the future (Liu *et al.*, 2012; Ostrowski and Schaub, 2012).

Second, we plan to study the extension of our approach to time-series data, although switching from a steady state to a dynamical viewpoint implies a growth of the search space. Fitting models whose steady states evolve between clearly separated time-scales (Terfve *et al.*, 2012) should be of similar complexity to the problem studied in this article. Fitting to the actual time-courses of a Boolean model has a higher level of complexity, as it requires to adjust the time-step of the Boolean model to the real time of the measurements.

More generally, we need to develop a rigorous framework to study models of biological networks as a family of plausible realizations, not of single networks. A first approximation could be to compare experimental data (ideally a distribution across individual cells) with a distribution of simulated results across a family of *single* logical models. The comparison of the distribution of feasible models with single cell data emerges as longer-term follow-up of this work that should provide deep insight into the cell-to-cell heterogeneity of signal transduction (Kolitz and Lauffenburger, 2012).

Altogether, we have implemented an open-source tool based on ASP providing a powerful framework to analyze networks models in systems biology. Further, several prospective tracks will certainly lead to future developments to extend and improve the functionalities of *caspo*.

## Supplementary Material

Supplementary Data
