# Supplementary material for: Exhaustively characterizing feasible logic models of a signaling network using Answer Set Programming
Source: Bioinformatics. 2013 Jul 12;29(18):2320–6. doi: 10.1093/bioinformatics/btt393 (PMC3753570; doi:10.1093/bioinformatics/btt393)
Supplement: Supplementary Data [file supp_29_18_2320__index.html]

Exhaustively characterizing feasible logic models of a signaling network using Answer Set Programming — Exhaustively characterizing feasible logic models of a signaling network using Answer Set Programming — Supplementary Data 

# Exhaustively characterizing feasible logic models of a signaling network using Answer Set Programming

## Supplementary Data

files

**Files in this Data Supplement:**

- Supplementary Data - pdf file
