# Supplementary material for: Exhaustively characterizing feasible logic models of a signaling network using Answer Set Programming
Source: Bioinformatics. 2013 Jul 12;29(18):2320–6. doi: 10.1093/bioinformatics/btt393 (PMC3753570; doi:10.1093/bioinformatics/btt393)
Supplement: Supplementary Data [file supp_btt393_supplementary.pdf]

# Exhaustively characterizing feasible logic models of a signaling network using Answer Set Programming: Supplementary Material

Carito Guziolowski* Santiago Videla* Federica Eduati Sven Thiele
Thomas Cokelaer Anne Siegel Julio Saez-Rodriguez

## Contents

**1 Learning Boolean logic models with ASP** **2**

**2 Performance** **4**

**3 Scalability** **4**

**4 Family of Optimal Models** **5**

**5 Family of Sub-optimal Models** **5**

## List of Figures

S1 Optimal logic models for HepG2 cells . . . 6
S2 Cross validation analysis . . . 6
S3 Distribution of models wrt MSEs for *caspo* and CellNOpt . . . 7
S4 Distribution of models with respect to Global Truth Tables for *caspo* and CellNOpt . . . 7
S5 Hierarchical clustering of GTTs found by *caspo* and CellNOpt . . . 8

## List of Tables

S1 Performance of *caspo* . . . 4
S2 Performance of CellNOpt . . . 4
S3 In silico benchmarks for *caspo* . . . 5
S4 Experiments to discriminate among 91 GTTs . . . 8

## List of Listings

S1 Logic program for learning Boolean logic models . . . 2
S2 Logic program to decide if 2 Boolean logic models are equivalent. . . . 3

# 1 Learning Boolean logic models with ASP

Recently, we proposed an ASP encoding addressing the problem of learning Boolean logic models. Experiments over *in silico* networks and datasets, have shown that ASP has excellent performances with respect to state-of-the-art approaches based on genetic algorithms. For this paper we use an extension of the previous encoding and we integrated in the introduced software tool *caspo*.

To illustrate how we represent our problem in ASP, first we give the following simplified example. Let $a \rightarrow c$ and $b \rightarrow c$, be two positive edges in the graph describing the given PKN. Then, we can derive the following hyperedges: $(\{a\},\{c\}),(\{b\},\{c\}),(\{a,b\},\{c\})$. This prior knowledge can be easily represented in ASP by the following set of logic predicates.

```
node(a). node(b). node(c). edge(a,1,c). edge(b,1,c). hyperedge(1,1,c). hyperedge(2,1,c).
hyperedge(3,2,c). in_he(1,a). in_he(2,b). in_he(3,a). in_he(3,b).
```

We use predicates `hyperedge(C,N,V)` to describe that the hyperedge $C$, has $N$ nodes in its source, and targets $V$. Further, we use predicates `in_he(C,V)` to describe that, $V$ is in the source of hyperedge $C$. Note that all possible hyperedges can be either given as an input, or generated from the graph during the grounding step (as we actually do).

Our problem consists of exploring the space of hypergraphs derived from a given graph, then one can generate every possible hypergraph $H$ by assuming each hyperedge as either present, or absent in $H$. That is, we need to *guess* which hyperedges are present, and which are absent. To this end, we use the following language construct.

```
{in_the_model(C,N,V)} :- hyperedge(C,N,V).
```

The logic rule above, is a non-deterministic construct describing that, each hyperedge $C$ with size $N$ and target $V$, can be either *in the model*, or not. Clearly, as the number of hyperedges increases, this gives rise to a combinatorial explosion of hypergraphs. A simple way to reduce the number of models is the following. Since $(a \wedge b) \vee a$ is logically equivalent to $a$, we say that a model having both hyperedges: $(\{a,b\},\{c\})$ and $(\{a\},\{c\})$ is redundant and must be discarded. Then, we can *check* whether a guessed model is redundant or not. We describe this by the following integrity constraint.

```
:- in_model(C1,M,V), in_model(C2,N,V), N < M, in_he(C1,U) : in_he(C2,U).
```

This rule eliminates every model having hyperedges $C1,C2$ such that, every node in the source of $C2$, is also in the source of $C1$.

Once we *guess and check* a model, we can easily compute, for every experimental condition (input), its Boolean predictions (outputs) using similar logic rules to the given above. Then, for each readout, we compare the experimental observations with the corresponding model prediction. Finally, we use the language construct `#minimize` to represent our lexicographic multi-objective function. First, assume that we generate predicates `residual(E,V,W)` to represent that, in the experiment $E$, the readout $V$ has a weighted mismatch $W$ with respect to the experimental observation. Then, if we want to find models minimizing the sum of residuals and size, we describe it with the following rules.

```
#minimize[in_the_model(_,N,_)=N @ 1].
#minimize[residual(_,_,W)=W @ 2].
```

With the last rules, ASP allows us to minimize weighted sum of literals guaranteeing global optimality among the valid solutions. We use `@ N` to describe the priority of each function in the lexicographic multi-objective optimization.

Next, the complete logic program used by *caspo* is shown in Listing S1.

Listing S1: Logic program for learning Boolean logic models

```
sub(set(U,S,nil),1,V)              :- edge(U,V,S).
sub(set(U,SU,set(W,SW,T)),N+1,V)  :- edge(U,V,SU), sub(set(W,SW,T),N,V), U < W.

in(U,S,set(U,S,T))    :- sub(set(U,S,T),N,V).
in(W,SW,set(U,SU,T)) :- in(W,SW,T), sub(set(U,SU,T),N,V).

{conjunction(C,N,V)} :- sub(C,N,V).
```

```
8  :- conjunction(C1,N,V), conjunction(C2,M,V), N < M, in(U,S,C2) : in(U,S,C1).
9
10 exp(E) :- exp(E,_,_).
11 fixed(E,V) :- exp(E), stimulus(V).
12 fixed(E,V) :- inhibitor(V), exp(E,V,0).
13
14 active(E,V) :- exp(E,V,1), stimulus(V).
15 active(E,V) :- exp(E), conjunction(S,M,V), not fixed(E,V),
16                active(E,U) : in(U,1,S), not active(E,U) : in(U,-1,S).
17 #const p = 2.
18 #const maxsize = -1.
19
20 dfactor(F) :- F = #pow(10, p).
21
22 :- maxsize + 1 [conjunction(_,N,_) = N], maxsize >= 0.
23
24 residual(D,V,1,#pow(F-D,2)) :- obs(E,V,D), dfactor(F), D<F.
25 residual(D,V,0,#pow(D,2))   :- obs(E,V,D), D>0.
26
27 #minimize[conjunction(_,N,_) = N @ 1].
28 #minimize[active(E,V) : obs(E,V,D) : residual(D,V,1,W) = W @ 2,
29          not active(E,V) : obs(E,V,D) : residual(D,V,0,W) = W @ 2].
```

Lines 1-5 generate every possible hyperedge by computing all subsets among the direct predecessors of each node. Line 7 defines, for each hyperedge the corresponding conjunction in the logic model as either present or absent (choice rule). Line 8 eliminates redundant models by checking inclusion between conjunctions. Line 10-16 describe the signal propagation under each experimental condition. Line 22 defines an optional constraint over the size of models. Lines 24 and 25 define the residual between predictions and observations. Finally, lines 27-29 define the multi-objective function.

Next, in the Listing S2 we show the logic program used to compute GTTs. Here, we search for any experimental condition generating at least one difference between 2 given models. Then, *caspo* run this program looking for equivalences among all models found during learning.

Listing S2: Logic program to decide if 2 Boolean logic models are equivalent.

```
1  model(I) :- conjunction(I,_,_,_).
2
3  {exp(V)} :- stimulus(V).
4  {exp(V)} :- inhibitor(V).
5
6  fixed(V) :- stimulus(V).
7  fixed(V) :- inhibitor(V), exp(V).
8
9  active(I,V) :- exp(V), stimulus(V), model(I).
10 active(I,V) :- conjunction(I,S,M,V), not fixed(V),
11                active(I,U)   : in(U,1,S),
12                not active(I,U) : in(U,-1,S).
13
14 diff :- active(I1,V), not active(I2,V), I1 != I2, readout(V), model(I1;I2).
15 :- not diff.
```

Lines 3 and 4 define every possible experimental condition, i.e. each stimulus or inhibitor can be present or absent. Lines 6-12 extend lines 11-16 in Listing S1 to propagate the signal in each model. Line 14 defines if there is at least one difference in the readouts of both models. Finally, line 15 defines an integrity constraint to decide if the models are equivalent or not. That is, whether exists an experimental condition to generate different output or not.

Table S1: Performance of *caspo*

| Tolerance (%) | # Models | Learning models time (sec) | # GTTs | Computing GTTs time (sec) |
|---|---|---|---|---|
| 0 | 16 | 0.3 (0.13) | 1 | 0.3 |
| 2 | 192 | 1.2 (0.15) | 4 | 5.7 |
| 4 | 918 | 5.5 (0.41) | 12 | 63.7 |
| 6 | 6048 | 36.8 (0.63) | 38 | 1285.5 |
| 8 | 7378 | 48.7 (0.77) | 60 | 2572 |
| 10 | 11700 | 70.3 (1.25) | 91 | 8286.4 |

Table S2: Performance of CellNOpt

| # Runs | # Models | Total time (hours) | Avg. time/run (sec) | Avg. time best model/run (sec) |
|---|---|---|---|---|
| 1000 | 4706 | 262 | 945 (std: 306) | 310 (std: 224) |

# 2 Performance

The performance of *caspo* over our case study[1] is shown in Table S1. For increasing MSE tolerance with respect to the optimal one (from 0% to 10%) we report: number of models found, learning models time, number of GTTs found and computation time for computing GTTs. To emphasize the solving power of ASP, in the learning time column, we report in parentheses the computation time when running the ASP solver (*clasp*) in quiet mode (only counting solutions without printing). Computations were run in a MacBook Pro, Intel Core i7, 2.7 GHz and 4 GB of RAM using *caspo 1.3* (*gringo* 3.0.3 and *clasp* 2.1). Note that computing GTTs, which could be computationally expensive for large number of models, is optional. The performance of CellNOpt over 1000 runs is shown in Table S2. We report the number of models found within 10% of tolerance, total computation time, average (and standard deviation) time per run to converge, and average (and standard deviation) time per run to find the best model. Computations were run on the EBI cluster with an average processors speed of 2.5 GHz. The CellNOpt version used in this analysis is 1.4.0 with the default parameters of the genetic algorithm except for the population size (100) and the maximum stall generation (1000). The times and number of models reported in the Table S2 can change significantly with the parameters used in the genetic algorithm. For example, reducing the elitim parameter increases the probability to find an optimal model.

# 3 Scalability

To test the scalability of our approach (time computation for the optimal network), we performed benchmarks on artificial networks of increasing size. Starting from an in silico PKN with 246 species, 9 benchmark models were generated with increasing numbers of nodes (from 31 to 240) and hyperedges (from 130 to 669) by selecting different sets of stimuli, inhibitors and readouts and connecting them with hyperedges taken from the extended PKN. These Boolean models were used to simulate in silico data using CellNOpt. Apart from the datasets simulated without noise, 2 additional datasets were generated for each model by adding a Gaussian noise with zero mean and standard deviation equal to 0.1 (corresponding to the level of noise shown in the real data) and 0.3 respectively. We show the computation time of caspo on these examples. For networks having up to 80 readouts and 202 possible hyperedges, caspo is able to solve the optimization problem in less than 8 seconds. Next, for 100 readouts the number of hyperedges increases significantly (from 202 to 338), leading to an increase in computation time (similarly, the same happens between 160 and 200 readouts: from 440 to 616 hyperedges). Moreover, for the larger benchmarks we have found that the introduced noise can have a significant impact for high values (0.3 in our case). For such cases, we can see a decrease of performance in 2 orders of magnitude or even reaching the time limit (10 hours). We note that this is a quite high value when compared with experimental techniques. For example, the luminex technology used in the real case of this paper has an error of $\sim 0.1$. For this error, the efficiency is very good (see Table S3).

Finally, we also report the computation time when using the unreleased solver unclasp[2]. We note that unclasp performs even better than clasp on our larger benchmarks. Moreover, we have found that a different encoding for

[1] `http://www.ebi.ac.uk/~cokelaer/cellnopt/data/ExtLiverPCB.html`
[2] `http://www.cs.uni-potsdam.de/wv/pdfformat/ankamasc12a.pdf`

Table S3: Performance of *caspo* (using *clasp*) and the unreleased solver (*unclasp*) on networks with increasing readouts and hyperedges for several levels of noise. We report CPU time in seconds using a timeout of 10 hours (36000s).

| | noise | | | | | |
|---|---|---|---|---|---|---|
| | 0 | | 0.1 | | 0.3 | |
| readouts (hyperedges) | clasp | unclasp | clasp | unclasp | clasp | unclasp |
| 31 (130) | 0.08 | 0.03 | 0.05 | 0.05 | 0.12 | 0.07 |
| 40 (149) | 0.64 | 0.05 | 0.51 | 0.12 | 2.06 | 0.13 |
| 60 (177) | 0.21 | 0.08 | 0.2 | 0.38 | 3.15 | 0.64 |
| 80 (202) | 3.7 | 0.19 | 7.81 | 0.56 | 3.87 | 1.24 |
| 100 (338) | 80.62 | 0.58 | 48.07 | 8.53 | 1162.83 | 491.24 |
| 120 (397) | 199.38 | 0.87 | 151.43 | 23.67 | 14005.73 | 53.45 |
| 160 (440) | 167.08 | 1.3 | 287.54 | 81.77 | 23229.12 | 1747.18 |
| 200 (616) | 1672.65 | 3.61 | 2188.31 | 638.95 | $> 36000$ | 1826.41 |
| 240 (669) | 4361.71 | 4.89 | 5107.75 | 1172.71 | $> 36000$ | 6460.67 |

the objective function could improve the performance of unclasp significantly. However, unclasp is under active development and no stable version has been released yet. Nevertheless, we hope to include this solver in a future version of caspo. Furthermore, the performance of caspo can be improved already by using the multi-threading version of clasp (not included by default due to license issues).

# 4 Family of Optimal Models

Both *caspo* and CellNOpt (combining multiple runs) found 16 global optimum solutions to our case study. The union of the 16 models is shown in Fig S1. The 16 different models arise due to four pairs of sub-models (modules) equivalent in terms of score. These modules represent alternative ways to activate specific nodes and are independent from each other. For each pair, only one of the modules appears in a given model; that is, they are *mutually-exclusive*. Thus, selecting either member of each pair provides an optimal model and all possible combinations give rise to the $2^4 = 16$ models.

**Cross-Validation:** To show the robustness of *caspo*, we have performed 10 runs of 10-fold cross-validation. That is, 10% of complete experiments (6 or 7 out of 64) was randomly removed and learning was performed from the remaining 90% in the dataset. We show in Fig. S2 the MSE of optimal models over the testing data. The median MSE among all runs was between 0.0430 and 0.0535, whereas the average was 0.0489. That is, almost the same compared to the MSE when using the complete dataset (0.0499).

# 5 Family of Sub-optimal Models

To illustrate the limitations of stochastic search, we have performed multiple runs of CellNOpt over the same case study described in the main text. From 1000 independent runs of CellNOpt, we found 4,706 different sub-optimal models whose MSE spanned from 0.0499 to 0.0543 (see Fig. S3). This family of models was found combining 20% of the runs, whereas in the other 80% all models found were out of the allowed tolerance range. Notably, the 16 optimal models (MSE=0.0499) were found by CellNOpt. In average, CellNOpt recovered around 50% of the models but with a high variability with respect to MSEs. For example, while 100% of models was found at MSE 0.0499 (optimum), only 2.8% of the models at MSE 0.053 were found.

Concerning GTTs, the 4,706 models exhibit 51 input-output behaviors out of the 91 we found with caspo (see Figs. S4 and S5). The genetic algorithm was able to find a relatively good sampling of GTTs. Nevertheless, 44% of the feasible input-output behaviors were not found.

**Discriminating among GTTs** In order to discriminate among 91 GTTs, we found that at least 7 experiments are required (Table S4). Note that these experiments are not the optimal experiments as described in the main text. While finding the “simplest” experiments maximizing pairwise differences, is relatively easy for a few GTTs (as shown in

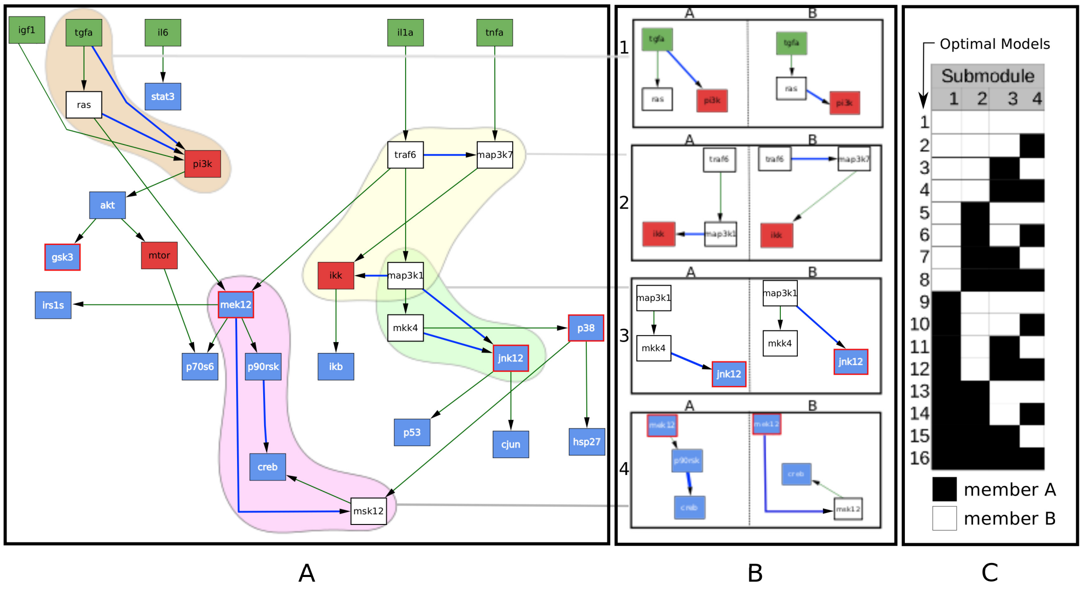


Figure S1: **Optimal logic models for HepG2 cells computed by *caspo*. (A)** Network of the union of 16 optimal models. Green nodes represent ligands that are experimentally stimulated. Red (or red-bordered) nodes represent those species that are inhibited with an small molecule inhibitor (drug). Blue nodes represent species that were measured using the Luminex technology. White nodes are neither measured nor perturbed. Green links represent hyperedges present in all models, and blue hyperedges present in some of the models. All models have logical gates of type OR. The colored regions highlight four pairs of mutually-exclusive modules. The network was generated with Cytocopter. **(B)** Mutually-exclusive modules. For the gates activating the nodes pi3k, ikk, jnk12, and creb, there are two alternative modules that are equivalent in terms of the value of the scoring function, denoted as A and B. **(C)** Distribution of the mutually-exclusive modules across the family of 16 optimal models. White or black boxes refer to member A or B in panel B.

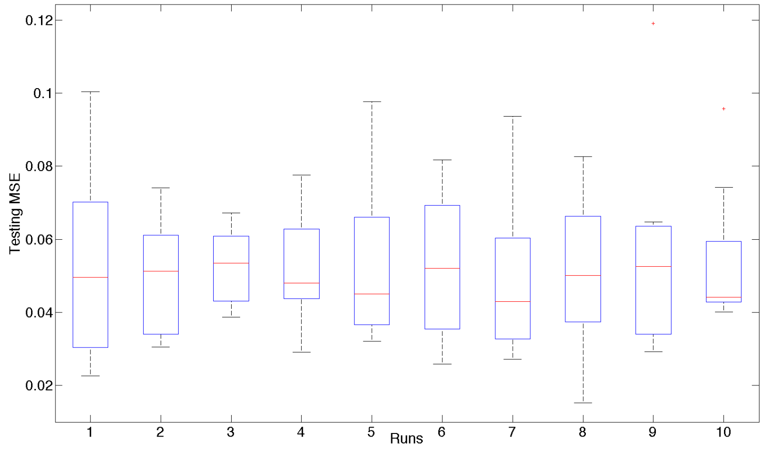


Figure S2: **Cross validation:** 10% of complete experiments (6 or 7 out of 64) was randomly removed and learning was performed from the remaining 90% in the dataset. There was no significant difference in MSE obtained using the complete dataset and the average of median MSEs among all cross-validation runs.

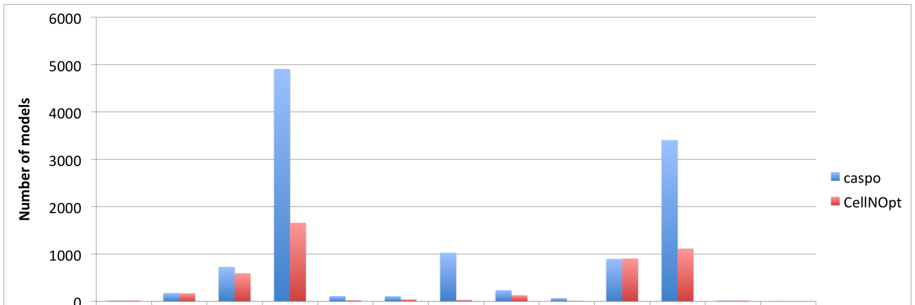


| | 0.0499 | 0.0507 | 0.051 | 0.0519 | 0.0522 | 0.0523 | 0.053 | 0.0531 | 0.0534 | 0.0539 | 0.0542 | 0.0543 | 0.0546 |
|---|---|---|---|---|---|---|---|---|---|---|---|---|---|
| caspo | 16 | 176 | 726 | 4910 | 112 | 108 | 1026 | 236 | 68 | 896 | 3406 | 16 | 4 |
| CellNOpt | 16 | 170 | 593 | 1660 | 22 | 39 | 29 | 129 | 11 | 906 | 1115 | 16 | 0 |

Figure S3: **Distribution of models wrt MSEs for caspo and CellNOpt.** Running caspo (blue) we found 11,700 models while from the multiple runs of CellNOpt (red) we found 4,706 models within the allowed tolerance range.

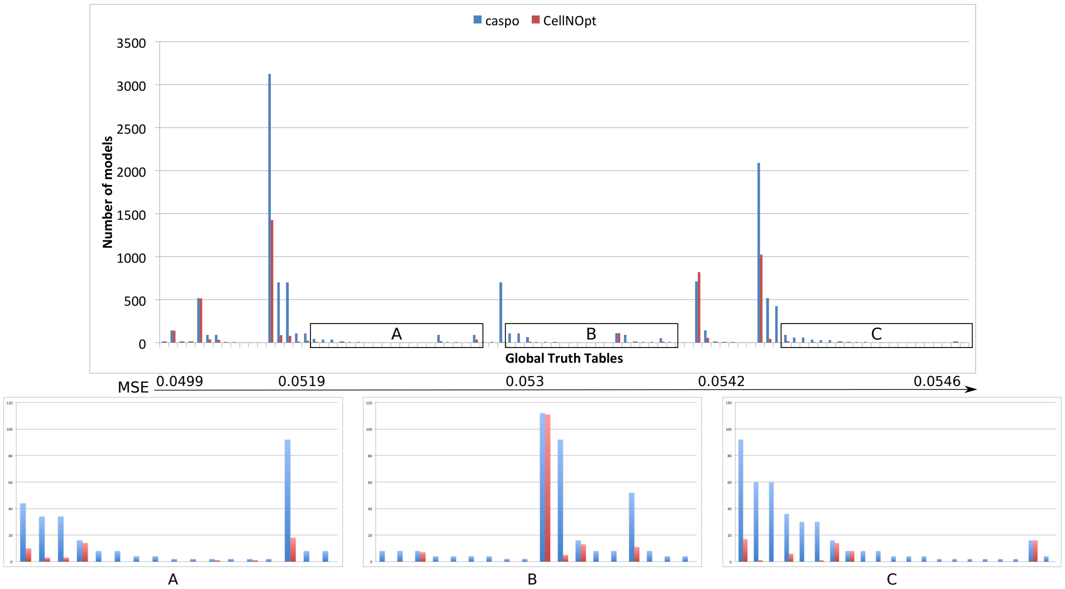


Figure S4: **Distribution of models with respect to Global Truth Tables for caspo and CellNOpt.** GTTs found by caspo (91 in blue) and CellNOpt (51 in red) considering 10% of tolerance. GTTs are ordered (from left to right) first according to their MSEs (from 0.0499 to 0.0546), and then according to their number of models. The number of different models grouped by the same GTT is plotted in vertical bars. A zoom is made over 3 blocks of GTTs (panels A, B and C) having low number of models to facilitate the reading.

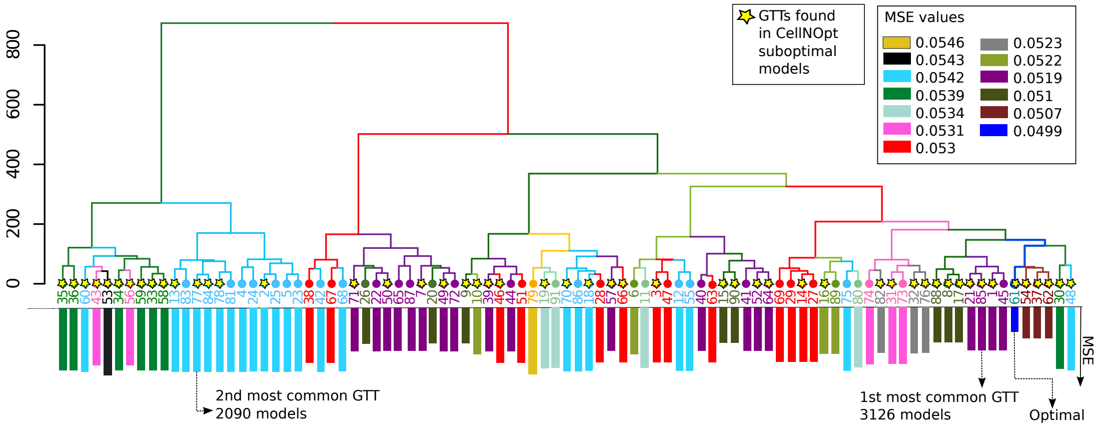


Figure S5: **Hierarchical clustering of GTTs found by caspo and CellNOpt.** Hierarchical clustering of the 91 GTTs based on their predictions for the readouts across all experimental conditions. Bars length on the leafs represents the corresponding MSE value for each GTT. The GTTs with stars correspond to the ones reported by CellNOpt over the 4,706 models.

Table S4: Experiments to discriminate among 91 GTTs

| IGF1 | TGFa | TNFa | IL1a | IL6 | mTORi | MEK12i | JNK12i | p38i | PI3Ki | IKKi |
|---|---|---|---|---|---|---|---|---|---|---|
| 1 | 0 | 1 | 0 | 0 | 1 | 0 | 0 | 1 | 0 | 0 |
| 0 | 1 | 1 | 0 | 0 | 0 | 0 | 0 | 0 | 1 | 0 |
| 0 | 1 | 0 | 1 | 0 | 0 | 1 | 0 | 0 | 1 | 1 |
| 1 | 0 | 0 | 1 | 0 | 0 | 0 | 1 | 0 | 0 | 0 |
| 0 | 1 | 1 | 0 | 1 | 0 | 1 | 0 | 1 | 0 | 0 |
| 0 | 0 | 1 | 1 | 1 | 0 | 1 | 1 | 0 | 0 | 0 |
| 1 | 1 | 0 | 1 | 0 | 0 | 0 | 0 | 1 | 0 | 0 |

the main text), doing the same over 91 GTTs (where 7 experiments are required) is a significantly larger optimization problem. Thus, we left such analysis for a future work.
